# Differentiating Coeliac Disease from Irritable Bowel Syndrome by Urinary Volatile Organic Compound Analysis – A Pilot Study

**DOI:** 10.1371/journal.pone.0107312

**Published:** 2014-10-16

**Authors:** Ramesh P. Arasaradnam, Eric Westenbrink, Michael J. McFarlane, Ruth Harbord, Samantha Chambers, Nicola O’Connell, Catherine Bailey, Chuka U. Nwokolo, Karna D. Bardhan, Richard Savage, James A. Covington

**Affiliations:** 1 Clinical Sciences Research Institute, University of Warwick, Coventry, Warwickshire, United Kingdom; 2 School of Engineering, University of Warwick, Coventry, Warwickshire, United Kingdom; 3 Department of Gastroenterology, University Hospital Coventry & Warwickshire, Coventry, Warwickshire, United Kingdom; 4 MOAC Doctoral Training Centre, University of Warwick, Coventry, Warwickshire, United Kingdom; 5 Department of Bioinformatics, University of Warwick, Coventry, Warwickshire, United Kingdom; 6 Rotherham General Hospital, Rotherham, Yorkshire, United Kingdom; University Hospital Llandough, United Kingdom

## Abstract

Coeliac disease (CD), a T-cell-mediated gluten sensitive enteropathy, affects ∼1% of the UK population and can present with wide ranging clinical features, often being mistaken for Irritable Bowel Syndrome (IBS). Heightened clinical awareness and serological screening identifies those with potential coeliac disease; the diagnosis is confirmed with duodenal biopsies, and symptom improvement with a gluten-free diet. Limitations to diagnosis are false negative serology and reluctance to undergo biopsy. The gut microbiome is altered in several gastrointestinal disorders, causing altered gut fermentation patterns recognisable by volatile organic compounds (VOC) analysis in urine, breath and faeces. We aimed to determine if CD alters the urinary VOC pattern, distinguishing it from IBS. 47 patients were recruited, 27 with established CD, on gluten free diets, and 20 with diarrhoea-predominant IBS (D-IBS). Collected urine was stored frozen in 10 ml aliquots. For assay, the specimens were heated to 40±0.1°C and the headspace analysed by Field Asymmetric Ion Mobility Spectrometry (FAIMS). Machine learning algorithms were used for statistical evaluation. Samples were also analysed using Gas chromatography and mass spectroscopy (GC-MS). Sparse logistic regression showed that FAIMS distinguishes VOCs in CD vs D-IBS with ROC curve AUC of 0.91 (0.83–0.99), sensitivity and specificity of 85% respectively. GCMS showed a unique peak at 4′67 found only in CD, not D-IBS, which correlated with the compound 1,3,5,7 cyclooctatetraene. This study suggests that FAIMS offers a novel, non-invasive approach to identify those with possible CD, and distinguishes from D-IBS. It offers the potential for monitoring compliance with a gluten-free diet at home. The presence of cyclooctatetraene in CD specimens will need further validation.

## Introduction

Coeliac disease is a T-cell mediated gluten sensitive enteropathy, affecting approximately 1% of the UK population, although only 10–15% of patients with the condition are diagnosed [1], [2]. It can be clinically difficult to distinguish from diarrhoea predominant Irritable bowel Syndrome (D-IBS); a non-inflammatory, multifactorial chronic condition affecting the GI tract [3]. The gold standard for diagnosis of coeliac disease is histopathological examination of small bowel biopsies, following initial serological investigations on patients in whom coeliac disease is suspected. Serological screening tests have been developed over the years and those currently in use are Anti-gliadin antibodies, anti-endomysial and anti-tissue transglutaminase (TTG) antibodies with the latter two being the most accurate [4], [5]. Anti endomysial tests showed a lower sensitivity than for dual (IgA and IgG) anti TTG antibodies (62–68% vs 90–92%) but a higher specificity (80–99% vs 81–83%). Combination testing of both endomysial and TTG antibodies has shown a slight increase in positive predictive value, negative predictive value and specificity, at the expense of sensitivity [6].

Both these serological tests however have had their accuracy questioned in young patients, the elderly and those with minimal mucosal damage. Furthermore their accuracy at monitoring response to a gluten free diet has also been debated [7], [8]. The value of these tests are further impaired in cases where the patients suffers from IgA deficiency and so the IgA antibodies that the tests would normally detect can be absent, leading to a false negative diagnosis [9].

The detection of specific patterns of volatile organic compounds (VOCs) in urine, breath, sweat and faeces has been a developing novel tool in recent years for the non-invasive detection of various disease states. The analysis of the VOCs pattern in patients breath using GCMS (Gas Chromatography and Mass Spectrometry) has been shown to distinguish not just cancer from non-cancer patients but also various cancer subtypes including lung, breast, prostate and colorectal cancer [10]. Furthermore analysis of VOCs in faeces has distinguished colorectal cancer from controls using Electronic nose (E-nose) technology [11]. VOCs patterns in urine have been analysed by E-nose and Field Asymmetric Ion Mobility Studies (FAIMS) and these have been able to distinguish between not only Inflammatory Bowel Disease (IBD) and healthy control patients but between patients with Crohn’s disease and ulcerative colitis and active disease from quiescent [12]. Patients with significant gastrointestinal side effects following pelvic radiotherapy have also been identified in this way [13]. More recently bile acid diarrhoea has been distinguished from ulcerative colitis and healthy controls using E-nose and FAIMs analysis of urine specimens [14]. For a detailed review on gas phase biomarkers in Gastroenterology, please see Arasaradnam et al [15].

VOCS have been found to be perturbed in many physiological and pathological states, including different diets and numerous disease states. The exact mechanism by which VOCs are generated is the subject of current research but their generation in the bowel is believed to be the result of dietary non-starch polysaccharides undergoing fermentation. As such, they represent the complex interaction of colonic cells, human gut microflora and invading pathogens [16], [17]. The resultant products of fermentation, which we have termed ‘the fermentome’ [14], [18], [19], [20] can exist in the gaseous phase and are present in exhaled air, sweat, urine and faeces [21]. Their presence in sweat, exhaled air and urine is presumed possible due to the altered gut permeability afforded in certain gut diseases [22]. We believe that VOCs represent a bio-signature that reflects the sum of the multifactorial influences (genetics, environmental factors including diet and disease states) affecting an individual.

The aim of our pilot study was to test the potential of FAIMS technology to differentiate patients with Coeliac disease from those with D-IBS using only urine samples.

## Materials and Methods

### 2.1 Subjects

47 patients were recruited prospectively for this study. The mean age was 48 years (SD 17) and there were 13 males. 27 patients had coeliac disease, confirmed histologically according to Marsh Criteria or according to HLA genotyping coupled with tTG serology. The Coeliac patients were established on gluten free diets at the time of urine specimen collection. Some patients were established on long term (10 years or more) gluten free diets and some were more recent diagnoses. tTG serology was performed on all the patients, either at initial screening or for monitoring in the long term patients. 20 patients had D-IBS according to the ROME II criteria with negative tTG serology, normal TSH as well as colonoscopy. These patients were selected as they were on diets inclusive of gluten. The demographics of the subjects are shown in table 1.

**Table 1:** Demographic data of subjects.

	coeliac disease	D-IBS
Number	27	20
Mean Age (SD)	53 (16)	42 (17)
Sex: M:F	7∶20	6∶14
Mean BMI (SD)	25 (4.4)	27 (6.6)
Current Smokers (% of whole population)	0% (0/47)	17% (8/47)
Alcohol: Greater than recommended for sex perweek (%of whole population)	11% (5/47)	4.3% (2/47)

### 2.2 Study Design

This was a case control study where patients were recruited from dedicated Gastroenterology outpatient clinics at University Hospital Coventry & Warwickshire, UK. Urine was then collected in standard universal sterilin specimen containers (Newport, UK) and frozen at −80°C for subsequent batch analysis, within 2 hours of collection.

### 2.3 Analysis

Urine samples were thawed by carefully raising the sample temperature to 5°C in a controlled procedure (usually done overnight) and then divided into separate 5 mL aliquots for analysis in each of the instruments employed in this study. The samples were aliquoted whilst still at this temperature to minimise loss of the chemical signal. One of these was transferred into a 20 mL glass vial by pipette and heated to 60°C to produce a reasonable headspace of volatiles. This headspace was extracted, mixed with a make-up flow of clean air at a ratio of 1∶3, and run through a Lonestar FAIMS (Owlstone Ltd.) using an attached ATLAS sampling unit and split flow box. The headspace of each sample was used to produce three full matrices of FAIMS data from the instrument, and blanks of clean, dry air were run both before and after each urine sample to ensure that the baseline response was returned. FAIMS is a process that separates and then measures the concentration of gases and vapours based on their different mobilities in high electric fields.

In addition, another 5 mL aliquot was pipetted into a 10 mL glass vial and sealed with a crimp lid for analysis using a Bruker Scion SQ gas chromatograph - mass spectrometer (GC-MS) fitted with a Restek Rxi-624Sil MS fused silica GC column (length 20 m, 0.18 mm internal diameter, 1.0 µm wall thickness) and a Combipal Autosampler (CTC, Switzerland) Due to the expected small concentrations of chemical components within the sample, the autosampler was improved by attaching a solid phase micro-extraction (SPME) pre-concentration fibre composed of poly-dimethylsiloxane (PDMS) of thickness 100 um. These sealed aliquots were individually heated to 60°C for 5 minutes, before the SPME fibre was introduced into the vials for a further 10 minutes to absorb the volatile organic compounds being released into the headspace above the urine. The now-saturated fibre was then heated to 250°C at the GC injector port to introduce the desorbed volatiles into the machine. Samples were mixed with helium carrier gas when entering the column at a split ratio of 1∶20 to maintain peak sharpness at the end detector. The GC oven followed a temperature programme for each sample in order to separate the constituent VOCs in terms of boiling point and molecular weight, by first holding at 50°C for 1 minute before increasing at a constant rate of 20°C/s up to a maximum of 280°C. The separated compounds were detected by chromatography, then fragmented and analysed by the mass spectrometer. Alternate 5 mL samples of de-ionised water were run through the system in between each urine sample, in order to verify that any VOCs identified were not introduced by the external environment.

### 2.4 Statistical Methods

In order to assess the FAIMS system's ability to differentiate between Coeliac disease and irritable bowel syndrome, we perform a leave-one-out cross-validation (LOO-CV), using several machine learning classification algorithms. Similar statistical methods have been used in previous studies [12].

LOO-CV is a technique for assessing our ability to make good predictions as to the disease class of an unseen sample. The method proceeds by training a classification algorithm on data from all-except-one of the samples. The algorithm is then used to predict the disease state of the held-out sample. Because the algorithm has no knowledge of the true disease state of this held-out sample, its prediction can be compared to the ground truth as a fair test of performance. This process is repeated in turn for each sample, so that we end up with a fair test of predictive ability across the whole data set. We repeat this procedure for each of the classification algorithms [23].

Before performing the LOO-CV, we apply some data pre-processing in order to better extract the signal from the data. We apply a 1D (Daubechies) wavelet transformation to the data vector from each sample, using the R package 'wavethresh'. Wavelets are a common method of data reduction used for audio compression [24]. We then remove all the wavelet coefficients whose variance across the data set is below a given threshold, on the basis that these will be dominated by noise. Finally, before training the classification algorithms, we use a Wilcoxon rank-sum test to find the most informative features as to disease state. We emphasise that this final step is performed inside the LOO-CV loop, and only on the training data, so that it cannot bias the results. The variance threshold and the number of features kept from the Wilcoxon analyses are parameters that have been tuned by hand to a limited degree in this analysis.

We considered three classification algorithms, all of which are known to give good performance for a wide range of tasks. It is important to consider several algorithms here, as some will typically be better suited to a given task than others. We use the following machine learning classification algorithms [25]:

- Sparse logistic regression: A version of logistic regression that imposes feature sparsity via an elasticnet prior. This has the effect of removing uninformative features from the analysis, thereby improving the quality of the analysis.
- Random Forest classification: An ensemble of decision trees, which leads to highly flexible data modelling.
- Support Vector Machine: A kernel-based method for separating the data space into separate disease subspaces.

### 2.5 Ethics

Scientific and ethical approval was obtained from local Research & Development Office as well as Warwickshire Ethics Committee ref: 09/H1211/38. Written informed consent was obtained from all patients who participated in the study.

## Results

The demographic data of the coeliac disease group and the D-IBS controls are described in Table 1. Details of the tTG titres and the Marsh classifications for the coeliac patients are shown in Table 2. A list of all drugs that the D-IBS and Coeliac patients were taking at the time of urine collection can be seen in Table 3.

**Table 2:** Tissue Transglutamase (TTG) titres at time of specimen collection and Marsh scores at diagnosis.

	TTG serology (at time of urine collection) kU/L				
Marsh Score (at diagnosis)	<1	1–4.9	5.0–9.9	10.0–14.9	15+
I			1*		
II	1				
IIIa	4	4			1
IIIb	3	1	2		
IIIc	4	2	1	1	

*HLADQ2+.

N.B. 2 patients did not have their Marsh scores available, both were established on long term gluten free diets and had a tTG titre of <1 kU/L at the time of urine collection.

**Table 3:** Drugs being taken by Coeliac and D-IBS patients at time of urine collection.

Medication		Coeliac Disease (n = 27)	D-IBS (n = 20)
Gastrointestinal tract	Antispasmodics (Mebeverine, Buscopan)	2.1% (1/47)	6.4% (3/47)
	Laxatives (Fybogel, Movicol, Lactulose)	2.1% (1/47)	8.5% (4/47)
	Anti-diarrhoea (Loperamide)	2.1% (1/47)	4.3% (2/47)
	Bile acid sequestrants (Ursodeoxycholic acid)	2.1% (1/47)	0% (0/47)
	5HT 4 agonist (Prucalopride)	0% (0/47)	4.3% (2/47)
	Proton Pump Inhibitors (omeprazole, lansoprazole)	0% (0/47)	14.9% (7/47)
	Anti-emetics (Domperidone)	0% (0/47)	2.1% (1/47)
	Multivitamins	0% (0/47)	6.4% (3/47)
Others	Iron	2.1% (1/47)	2.1% (1/47)
	Antidepressants (SSRIs, TCAs)	11% (5/47)	13% (6/47)
	Bisphosphonates	19% (9/47)	0% (0/47)
	Calcium & Vitamin D supplementation	19% (9/47)	0% (0/47)
	Thyroxine	4.3% (2/47)	0% (0/47)
	Antibiotics (trimethoprim/nitrofurantoin)	2.1% (1/47)	0% (0/47)
	Opioids	4.3% (2/47)	4.3% (2/47)
	Antihistamine	4.3% (2/47)	0% (0/47)
	Hormone replacement therapy/COCP	6.4% (3/47)	0% (0/47)
	Warfarin	2.1% (1/47)	0% (0/47)
	Antipsychotics	0% (0/47)	2.1% (1/47)
	Pregabalin	2.1% (1/47)	0% (0/47)
	Monteleukast	2.1% (1/47)	0% (0/47)
Cardiovascular	Anti-hypertensives	6.4% (3/47)	2.1% (1/47)
	Statins	6.4% (3/47)	4.3% (2/47)
	Diuretics	2.1% (1/47)	0% (0/47)
	Aspirin	2.1% (1/47)	0% (0/47)
Diabetic	Insulin	2.1% (1/47)	0% (0/47)
	Oral hypoglycaemics	2.1% (1/47)	0% (0/47)

### FAIMS

**Figure 1:**
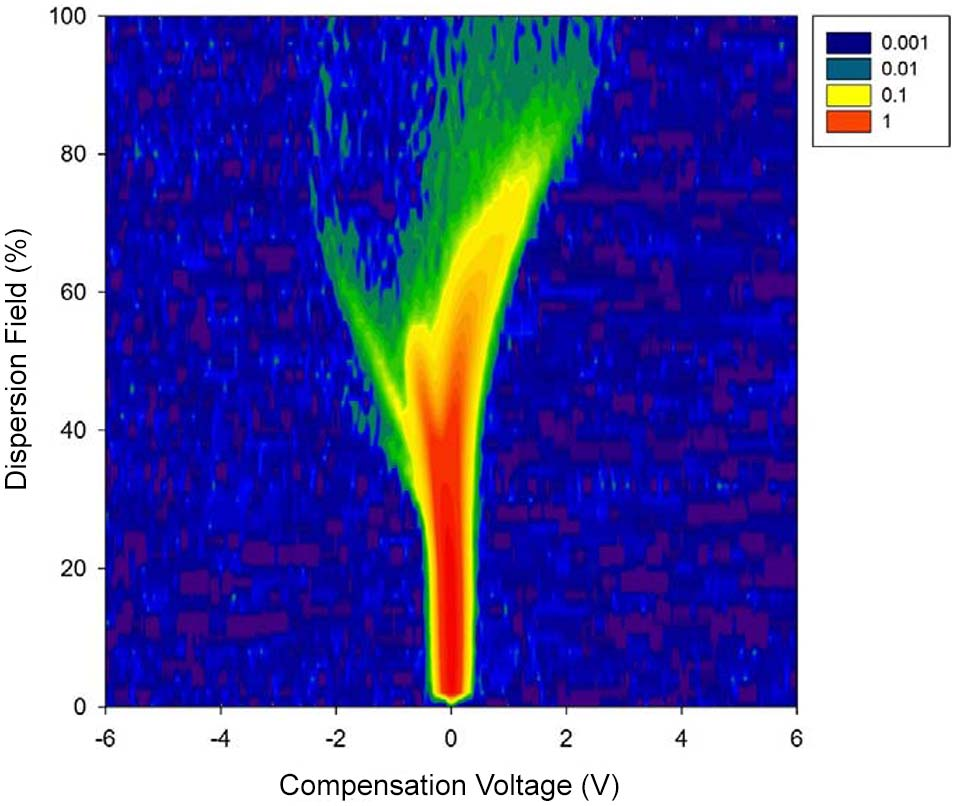
Raw FAIMS output for a Coeliac patient urine sample.

The analysis of the FAIMS data for coeliac patients and controls was carried out using three different machine learning classifiers, as described above. Figure 1 shows a raw plot of the data created by FAIMS technique. As mobility of a chemical is not constant and is a function of applied electric field, the instrument scans through a range of different settings (which is described by the dispersion field in figure 1), with the compensation voltage being a fixed DC voltage that compensates for the mobility of the molecule, allowing gas/vapour molecules with only that specific mobility to be measured.

The results are shown in Table 4. The best performance was obtained using sparse logistic regression, with a ROC curve AUC of 0.91 (0.83–0.99), Sensitivity of 0.85 (0.66–0.96), and Specificity of 0.85 (0.62–0.97).

**Table 4:** Results of the machine learning analysis.

Method	AUC	Sensitivity	Specificity
Sparse Logistic Regression	**0.91** (0.83–0.99)	**0.85** (0.66–0.96)	**0.85** (0.62–0.97)
Random Forest	0.81 (0.68–0.93)	0.78 (0.58–0.91)	0.80 (0.56–0.94)
Support Vector Machine	0.87 (0.77–0.97)	0.78 (0.58–0.91)	0.80 (0.56–0.94)

Shown here are the ROC curve Area-Under-Curve (AUC) scores, sensitivities and specificities for three classification algorithms. The values are computed using a leave-one-out cross-validation. The 95% confidence intervals are shown in brackets. The sensitivities and specificities are determined from the ROC curve, selecting in each case a threshold that gives good values for both.

**Figure 2:**
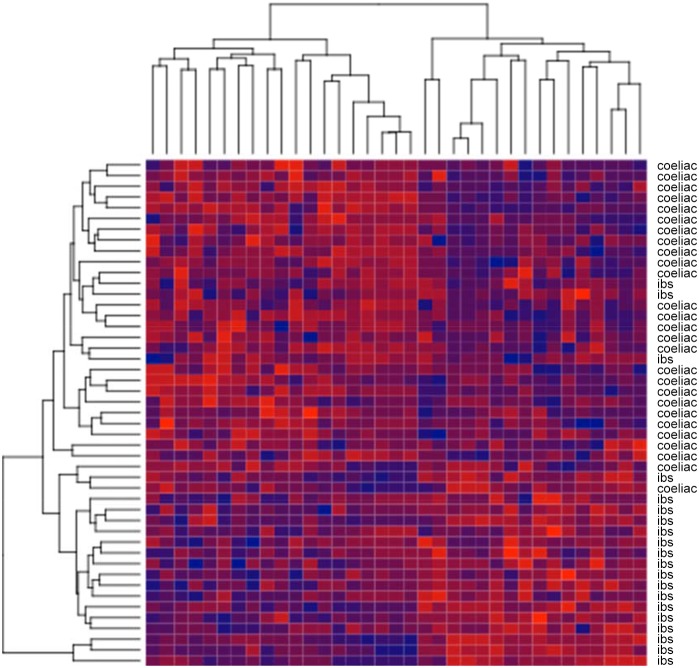
LDA FAIMS analysis. Heatmap showing the selected FAIMS features from one of the CV folds. The data are clustered, showing that there is a clear difference between the coeliac disease and D-IBS.

Figure 2 shows a heat map of the FAIMS features identified as informative (where each line refers to the same features of from one sample). As can be seen, there is a clear difference in the data signatures between coeliac and D-IBS patients. This signature leads to the strong predictive performance of the machine learning algorithms.

**Figure 3:**
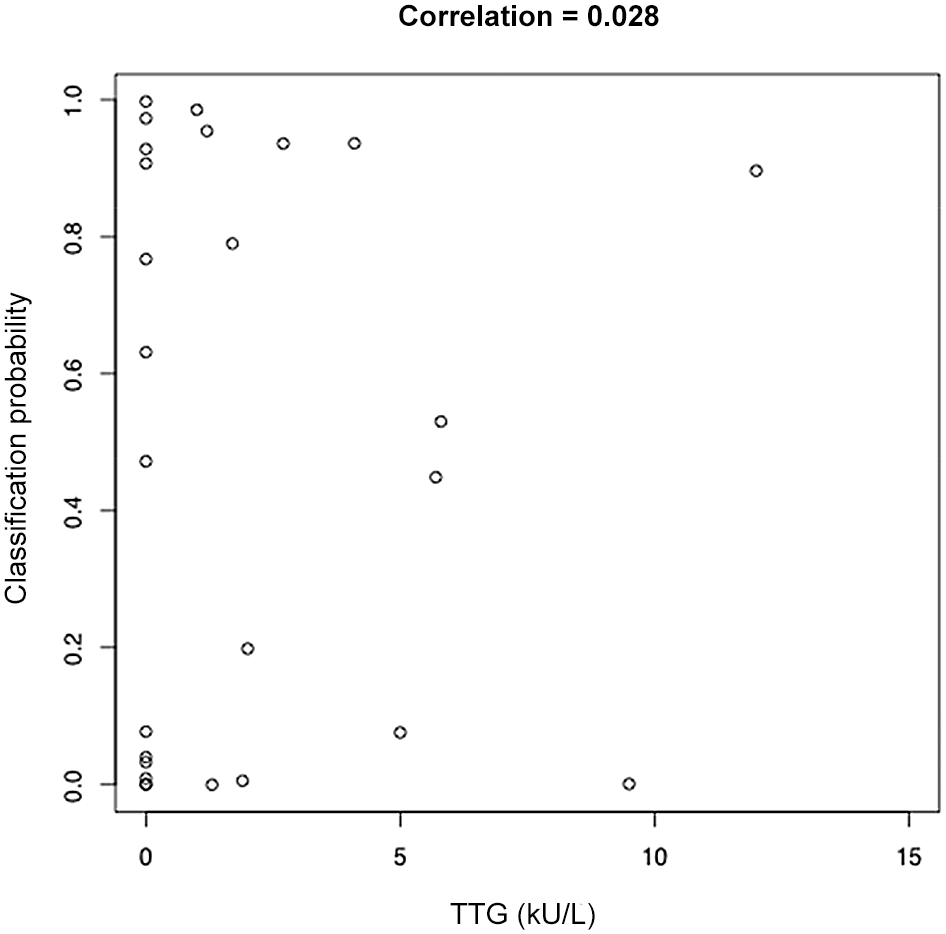
Scatter plot of TTG serology vs classification probability for Coeliac cases. The classification probabilities are the probability of a given patient having Coeliac disease, as determined by the sparse logistic regression algorithm. The overall correlation of these points is 0.28. One outlying point with TTG>60 kU/L has been removed.

Table 5 and Figure 3 show comparisons between the classification probabilities and (respectively) Marsh score, and TTG serology. As can be seen, within this data set there are no strong relationships between the probability of having coeliac (as determined by sparse logistic regression) and either Marsh score or TTG serology.

**Table 5:** Predicted probability of coeliac disease and Marsh scores at diagnosis.

	Classification – probability of coeliac disease (sparse logistic regression)				
Marsh Score(at diagnosis)	0–0.2	0.2–0.4	0.4–0.6	0.6–0.8	0.8–1
I	1*				
II	1				
IIIa	3	1		2	3
IIIb	3		1		2
IIIc	2		2		4

*HLADQ2+.

N.B. two patients did not have their Marsh scores available.

**Figure 4:**
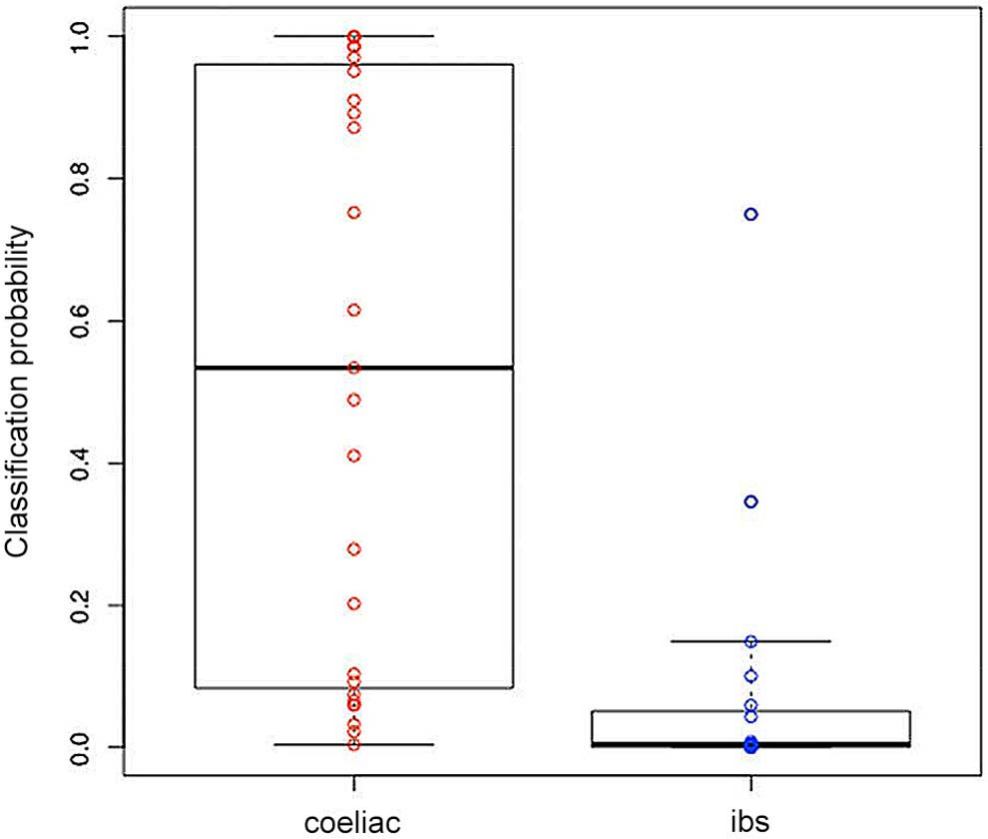
Classification probabilities by disease group. Box-and-whisker plot of the classification probabilities generated by sparse logistic regression. The boxes show the interquartile ranges. The whiskers show the data range, but are truncated to a maximum of twice the interquartile range.

Figure 4 shows the classification probabilities generated by sparse logistic regression, plotted by disease group.

### GC-MS

The data from the GC-MS were analysed by observing the retention times of chromatogram peaks, and comparing the corresponding mass spectra found by the instrument to those from a known NIST library of chemical components. This comparison comprises a measure of both forward- and reverse- matching between observed and known spectra which produce a list, ranked by probability, of potential chemical compounds that could have caused each peak.

In order to discover the most likely VOCs that make up a urine headspace sample, the highest-probability matching compounds for peaks at the same GC retention times were tallied for all samples and the most common were suggested as the probable source. Only clear peaks above the 1.8 MCps (microporous co-ordination polymers) threshold were identified, to ensure that the signals were significantly above the noise floor of the instrument. We identified over 70 separate chemicals, but there was a high variation in individual sample composition, but a number of VOCs were found to be present in urine samples with a significant degree of certainty. Table 6 lists the GC peaks found in the majority of urine samples along with their retention times, associated mass spectra peaks, and highest probability NIST library ‘hits’.

**Table 6:** Mass Ion Peaks and NIST Identifications of GC Peaks.

GC Retention Time(mins)	Mass Ion Peaks (in order of relativeabundance)	NIST Identifications
5.30	133, 40, 44, 151, 150, 63	4-ethylbenzoic acid ester
7.63	44, 39, 40, 89, 52, 50, 75	Chloro/4-tertbutyl amphetamine
7.95	82, 54, 40, 43, 39, 93, 91	Carvone
9.76	191, 192, 44, 41, 57, 91, 206	2,4–bis (dimethylethyl) - phenol

**Figure 5:**
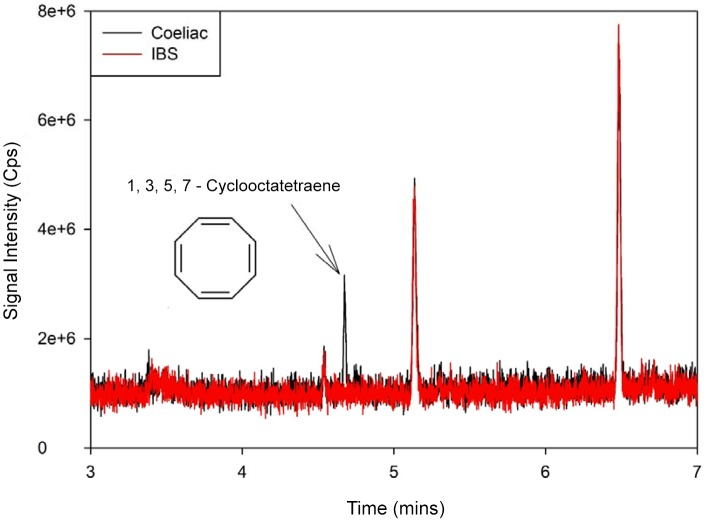
Section of coeliac disease sample chromatogram showing unique peak.

**Figure 6:**
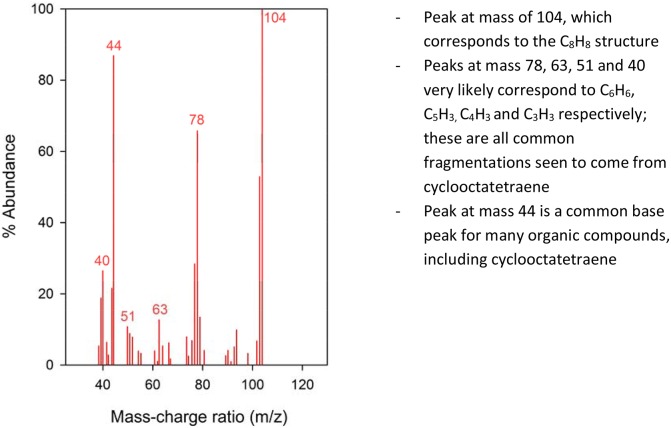
Section of mass spectrum corresponding to unique GC peaks. Peak at mass of 104, which corresponds to the C_8_H_8_ structure. Peaks at mass 78, 63, 51 and 40 very likely correspond to C_6_H_6,_ C_5_H3, C_4_H_3_, and C_3_H_3_ respectively; these are all common fragmentations seen to come from cyclooctatetraene. Peak at mass 44 is a common base peak for many organic compounds, including cyclooctatetraene.

Notably, one of the compounds discovered using this method was observed at approximately 4.67 minutes in the chromatograms of the samples taken from coeliac disease patients, while being absent in those of D-IBS sufferers (Figures 5 and 6). The compound with the mass spectrum that is by far the most consistent with those observed here is 1, 3, 5, 7 cyclooctatetraene, as shown in Figure 5. The mass spectra of an example GC peak from one of the coeliac samples are shown in Figure 6, illustrating the mass ratios of the major components found in this region. Each of these components has subsequently been checked against commonly-known fragments with the same ratio; Figure 6 suggest potential fragments from cyclooctatetraene that correspond to each individual MS peak from the samples. However, validation of this chemical using standards of 1, 3, 5, 7 cyclooctatetraene at a specified range of concentrations is still required to validate its presence within the urinary headspace. The raw data from these experiments are available on request to the authors.

## Discussion

Our pilot study provides initial evidence that FAIMS has potential application as an alternative non-invasive test for the initial screening of patients suspected of having coeliac disease. This is done via the detection of a unique gas phase bio-odorant fingerprint found in the urine of patients with coeliac disease. This expands on previous research which has shown that E-nose and FAIMS analysis can analyse and distinguish the VOCs patterns in urine of patients with UC, Crohn’s disease, bile acid diarrhoea, IBS and healthy controls (15).

The FAIMS data for the coeliac patients showed tight clustering and high reclassification accuracy, suggesting a discernable VOC profile. With suitable feature extraction, coeliac patients and IBS patients could be separated by FAIMS with a sensitivity and specificity of 85% respectively. IBS tends to be diagnosed in patients with diarrhoea, constipation or abdominal discomfort for which no underlying cause can be ascertained. Therefore, instead of a distinct VOC profile, there is likely to be large patient-to-patient variation, and this is reflected in the data found here.

Additionally, data given by the GC-MS has revealed a peak unique for those with coeliac disease – specifically mass spectra that indicate it is likely due to the volatile compound Cyclooctatetraene. Previous studies have shown production of this compound by various species of fungi for its inhibitory effect on the growth of other microbes [26], [27]. There have also been a number of studies into volatiles produced from stool samples [28], without being linked to any particular disease.

E-nose and FAIMS technology has been shown not only to distinguish UC from Crohn’s disease but also to differentiate active disease from patients in remission [12]. This could indicate a potential role for these technologies in the monitoring of compliance with a gluten free diet in coeliac patients as currently tTG antibodies have shown inconsistent results when used for this purpose [7], [8]. Analysis of the VOCs in urine could in the future represent a more effective and real time means of monitoring compliance by patients at home (with a portable device or specialised mobile phone application).

The unique chemical fingerprint produced by the different disease states shows the potential of this technology as an initial alternative screening test for coeliac disease. Furthermore it has the potential to aid in the further investigation of individuals with other GI disease in whom the diagnosis is not clear. VOCs are believed to be produced by colonic fermentation: the result of a complex interaction between the colonocyte cells, human faecal flora, mucosal integrity and invading pathogens [19]. These thereafter pass into bodily fluids and as a result, VOCs found in urine, faeces and breath have huge potential as biomarkers to aid in the assessment of gastrointestinal diseases. Any changes found in the pattern of VOCs are reflective of changes and variations within the gastrointestinal environment. This suggests a possible role for gut microflora dysbiosis in the pathophysiology of coeliac disease which has been found in several studies including paediatric coeliac disease [29], [30], [31], [32].

GCMS data also identified a chemical that could be correlated to the Coeliac disease state, with a high proportion of NIST library ‘hits’ suggesting 1, 3, 5, 7 Cyclooctatetraene. In addition, identification of this chemical was made via the NIST library by forward and reverse matching scores between documented spectra and those found in the sample set. However, further validation of the presence of this chemical is required using appropriate standards. Moreover, it is likely that there are additional bio-markers and we will be able to identify global changes in the total chemical profile. Future work will attempt to validate the chemicals identified here and to undertake a more thorough characterisation of the urinary headspace.

This pilot study serves to demonstrate the potential of IMS technology (FAIMS) using only urine samples to differentiate coeliac disease from other overlap gastrointestinal conditions such as IBS. Its advantages include portability, rapid real time and cost effective diagnostic approach. Further validation studies are necessary to confirm its accuracy as well as ability to distinguish between inflammatory and non-inflammatory conditions.
